# Preterm infant circulating sex steroid levels are not altered by transfusion with adult male plasma: a retrospective multicentre cohort study

**DOI:** 10.1136/archdischild-2021-323433

**Published:** 2022-03-01

**Authors:** Anders K Nilsson, Gunnel Hellgren, Ulrika Sjöbom, Andreas Landin, Henrik Ryberg, Dirk Wackernagel, David Ley, Ingrid Hansen Pupp, Matti Poutanen, Claes Ohlsson, Ann Hellstrom

**Affiliations:** 1 Department of Clinical Neuroscience, Institute of Neuroscience and Physiology, University of Gothenburg Sahlgrenska Academy, Gothenburg, Sweden; 2 Institute of Biomedicine, University of Gothenburg Sahlgrenska Academy, Gothenburg, Sweden; 3 Institute of Health and Care Sciences, University of Gothenburg Sahlgrenska Academy, Gothenburg, Sweden; 4 Department of Internal Medicine and Clinical Nutrition, Institute of Medicine, University of Gothenburg Sahlgrenska Academy, Gothenburg, Sweden; 5 Department of Clinical Chemistry, Sahlgrenska University Hospital, Gothenburg, Sweden; 6 Department of Neonatology, Karolinska University Hospital, Stockholm, Sweden; 7 Department of Clinical Science CLINTEC, Intervention and Technology, Karolinska Institutet, Stockholm, Sweden; 8 Department of Pediatrics, Institute of Clinical Sciences Lund, Lund University Hospital, Lund, Sweden; 9 Institute of Biomedicine, Research Centre for Integrative Physiology and Pharmacology and Turku Center for Disease Modeling, University of Turku, Turku, Finland; 10 Centre for Bone and Arthritis Research, Sahlgrenska Osteoporosis Centre, Department of Internal Medicine and Clinical Nutrition, Institute of Medicine, University of Gothenburg Sahlgrenska Academy, Gothenburg, Sweden; 11 Department of Drug Treatment, Sahlgrenska University Hospital, Gothenburg, Sweden

**Keywords:** endocrinology, intensive care units, neonatal, neonatology

## Abstract

**Objective:**

To determine if plasma transfusions with male donor plasma to very preterm infants affect circulatory levels of sex steroids.

**Design and patients:**

Retrospective multicentre cohort study in 19 infants born at gestational age <29 weeks requiring plasma transfusion during their first week of life.

**Setting:**

Three neonatal intensive care units in Sweden.

**Main outcome measures:**

Concentrations of sex steroids and sex hormone-binding globulin (SHBG) in donor plasma and infant plasma measured before and after a plasma transfusion and at 6, 12, 24 and 72 hours.

**Results:**

The concentrations of progesterone, dehydroepiandrosterone and androstenedione were significantly lower in donor plasma than in infant plasma before the transfusion (median (Q1–Q3) 37.0 (37.0–37.0), 1918 (1325–2408) and 424 (303–534) vs 901 (599–1774), 4119 (2801–14 645) and 842 (443–1684) pg/mL), while oestrone and oestradiol were higher in donor plasma (17.4 (10.4–20.1) and 16.0 (11.7–17.2) vs 3.1 (1.1–10.2) and 0.25 (0.25–0.25) pg/mL). Median testosterone and dihydrotestosterone (DHT) levels were 116-fold and 21-fold higher in donor plasma than pre-transfusion levels in female infants, whereas the corresponding difference was not present in male infants. Plasma sex steroid levels were unchanged after completed transfusion compared with pre-transfusion levels, irrespective of the gender of the receiving infant. The SHBG concentration was significantly higher in donor than in recipient plasma (22.8 (17.1–33.5) vs 10.2 (9.1–12.3) nmol/L) before transfusion but did not change in the infants after the transfusion.

**Conclusions:**

A single transfusion of adult male plasma to preterm infants had no impact on circulating sex steroid levels.

## Abstract (precis)

Sex hormones delivered via plasma transfusions from adult male donors to very preterm infants do not alter the circulating plasma concentrations in the infant, irrespective of gender.

**What is already known on this topic?**

- Very preterm infants often receive plasma transfusions within their first weeks of life.
- In most neonatal care settings, donor plasma for transfusion is exclusively derived from male adults

**What this study adds?**

- The sex steroid profile of male adult donor plasma differs substantially from that of preterm infant plasma.
- Preterm infant circulating sex steroid levels are similar before and after a transfusion with adult male plasma

**How this study might affect research, practice or policy?**

- More studies are needed to clarify the metabolic fate of the donor plasma steroids in the preterm infant and their possible effect on peripheral tissues.

## Introduction

Fresh frozen plasma is frequently given to preterm infants at the neonatal intensive care unit (NICU), but with large regional differences.^1–3^ Up to 15% of the infants admitted to NICUs received one or more plasma transfusions when all birth weights and birth gestational ages (GAs) are included,^4^ with higher frequency among the more immature infants.^1 5^ Indications for the plasma transfusions vary, with the perceived risk of bleeding with abnormal coagulation values, arterial hypotension or affected peripheral circulation as common reasons.

Blood products used for transfusion to neonates are derived from adult donors. As there is a risk for immune-mediated transfusion reactions when plasma from female donors with a history of pregnancy is used,^6 7^ in most care settings, only male donor plasma is considered for transfusion. Plasma is a complex matrix, containing a diversity of bioactive compounds with varying concentrations, depending on age and gender, among other factors. Little is known about the potential impact of factors supplied from adult male donors to the neonate during plasma transfusions.

The fetoplacental unit is untimely broken after preterm birth, thereby interrupting the transfer of sex steroids, steroid precursors and the intricate signalling between mother and fetus. The fetal adrenal cortex is a major site for steroid hormone synthesis during pregnancy, regulating intrauterine homeostasis and stimulating organ maturation.^8^ Maturity of the fetal adrenal cortex is related to GA, and preterm infants display a reduced capacity of adrenal steroid synthesis at birth^9 10^ coupled to an altered ability for extrauterine adaptation.^11 12^

Considerable fractions of androgens and oestrogens in the blood are bound to sex hormone-binding globulin (SHBG). In the SHBG-bond state, these hormones, including testosterone and oestradiol, are thought to be biologically inactive. Although SHBG levels are significantly lower in fetal serum compared with maternal, the fetal serum concentration of SHBG exceeds that of testosterone, oestradiol and dihydrotestosterone (DHT).^13^ It has been hypothesised that fetal SHBG can protect female fetuses from exposure to androgens and could also play a role in male sex differentiation by modulating the pool of active androgens.^14^

The effect on preterm infant plasma levels of sex steroids following transfusions is unknown. Hypothetically, excess sex steroids from plasma transfusion could alter infant levels and impose an endocrine effect on these susceptible patients. Furthermore, exogenous steroids supplied via transfusions may compete with endogenous steroids produced by the fetal zone for steroid receptor binding.^15^ One case report described a female neonate that developed clitoromegaly during the second month of life after repeated whole blood transfusions from her father.^16^ The infant was found to have high plasma testosterone levels, and the authors suggested that the masculinisation effect was attributed to the transfusions.

This study aimed to investigate if transfusion with male adult plasma affects circulatory sex steroid levels in receiving preterm infants.

## Methods

### Study cohort

Infants were recruited at the NICU at three centres in Sweden: Queen Silvia Children’s Hospital at the Sahlgrenska University Hospital in Gothenburg (n=10), Astrid Lindgren Children’s Hospital Huddinge at the Karolinska University Hospital in Stockholm (n=1) and Skåne University Hospital in Lund (n=8). Inclusion criteria were GA at birth <30 weeks and a postnatal age of <10 days. Exclusion criteria were previous plasma transfusion and severe malformations. Study participants were recruited between November 2015 and November 2016. Donor plasma was administered through a venous catheter during 30–120 min at a dose of 10–15 mL/kg according to clinical routines.

### Sample collection

EDTA plasma samples were collected immediately before the start and immediately after the completed transfusion, and then at 6, 12, 48 and 72 hours after the transfusion. If an additional transfusion started within 72 hours after the first transfusion, a blood sample was taken 72 hours after the end of the second transfusion. Within 1 hour after collection, samples were centrifuged 10 min at 1500 g and the plasma was stored in 0.5 mL cryotubes at −80°C. A portion of plasma (2 mL) was collected from donor plasma units and stored together with the infant samples. All samples were thawed and refrozen once before analysis. The number of samples analysed for sex steroids and SHBG according to time point and gender is presented in online supplemental table 1.

10.1136/fetalneonatal-2021-323433.supp1 Supplementary data

### SHBG and sex steroid analysis

Plasma sex steroids were analysed by a validated gas chromatography-tandem mass spectrometry (GC-MS/MS) method with a lower limit of quantification (LLOQ) for oestradiol, oestrone, testosterone, DHT, progesterone, androstenedione and dehydroepiandrosterone (DHEA) of 0.5, 0.5, 8, 2.5, 74, 12 and 400 pg/mL, respectively. Between 20 and 200 µL plasma were used for the analysis. Details of the methods used for the steroid analysis have been previously described.^17^

To calculate the total amount of testosterone present in infant blood plasma, infant plasma volume was assumed to be 50 mL/kg body weight.

SHBG measurements were performed in a subpopulation of infants using the Human SHBG Quantikine ELISA (cat: DSHBG0B, Bio-Techne, UK) according to the manufacturer’s instructions. Plasma samples were diluted at 1:75 and assayed as duplicates. Absorbance was measured at 450 nm with wavelength correction at 620 nm (Multiscan FC 3.1, Thermo Fisher Scientific). Sample concentrations were determined by comparison to a 7-point calibration curve with 4-parametric curve fit (R^2^ >0.99) using SoftMax Pro Software V.4.6 (Molecular Devices). The inter-assay coefficient of variation (CV) was 9.9% at 86 nmol/L and the maximum CV for duplicate samples was 16.7%.

### Statistical analyses

Statistical analyses were performed using IBM SPSS Statistics, V.27 (IBM Corp, Armonk, New York, USA) and all figures were made with R (The R Foundation for Statistical Computing, Vienna, Austria) and the ggplo2 package. Differences between groups were compared using the non-parametric Mann–Whitney U-test. Differences between time points with related samples were investigated using Wilcoxon signed-rank test. Steroid measurements below the LLOQ were set to LLOQ/2 for statistical analyses. For all tests, p values <0.05 were considered significant.

## Results

### Study cohort

The clinical characteristics of the study cohort are reported in online supplemental table 2. Nineteen infants with a median (min, max) GA at birth of 25.3 (22.9–28.9) weeks were included in the study. All infants received their studied plasma transfusion within 5 days after birth. The median (min, max) volume of plasma transfused was 9.0 (6.0–15.0) mL/kg. Within 72 hours after completing the studied transfusion, eight infants received a second plasma transfusion and one infant received a third plasma transfusion.

### Sex steroids in donor plasma and infant plasma before start of transfusion

**Figure 1:**
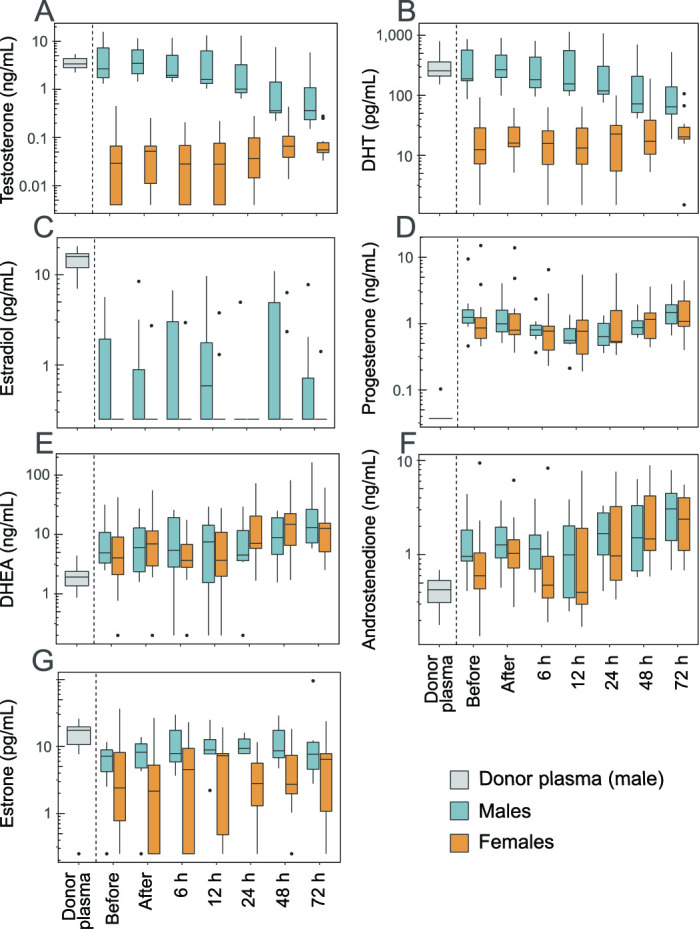
Sex steroids in donor plasma and infant plasma. Sex steroids were analysed in donor plasma and infant plasma just before and after completed plasma transfusion, and then followed 72 hours after the end of the transfusion. (A) Testosterone; (B) dihydrotestosterone (DHT); (C) oestradiol; (D) progesterone; (E) dehydroepiandrosterone (DHEA); (F) androstenedione; (G) oestrone. Boxes show median and IQR, whiskers the largest/smallest value within 1.5 times of the IQR, and outliers are individually plotted. Note that the y-axes are on the logarithmic scale.

Before transfusion start, the plasma concentration of three of the analysed sex steroids differed between male and female infants: testosterone, DHT and oestradiol were significantly lower in females than in males (figure 1 and table 1). Therefore, these steroids were analysed separately for males and females.

**Table 1:** Concentrations of sex steroids in infant plasma and donor plasma units before transfusion start

Sex steroid	Donor (n=19)	All infants (n=19)	Boys (n=7)	Girls (n=12)	P, boys vs girls	P, donor vs all	P, donor vs boys	P, donor vs girls
Progesterone (pg/mL)	37.0 (37.0–37.0)	901 (599–1774)	1237 (901–1997)	859 (599–1602)	0.205	<0.001	n.t.	n.t.
Dehydroepiandrosterone (pg/mL)	1918 (1325–2408)	4119 (2801–14 625)	4912 (2801–14 625)	4031 (1308–13 031)	0.672	0.002	n.t.	n.t.
Androstenedione (pg/mL)	424 (303–534)	842 (443–1684)	957 (842–1969)	599 (414–1189)	0.176	0.002	n.t.	n.t.
Testosterone (pg/mL)	3394 (2671–4509)	–	2668 (1650–11 495)	29.2 (4.0–70.7)	0.000	n.t.	0.565	<0.001
Dihydrotestosterone (pg/mL)	255 (208–361)	–	188 (171–856)	12.4 (6.7–30.5)	0.001	n.t.	0.565	<0.001
Oestrone (pg/mL)	17.4 (10.4–20.1)	3.1 (1.1–10.2)	7.2 (2.5–10.2)	2.4 (0.47–10.8)	0.373	<0.001	n.t.	n.t.
Oestradiol (pg/mL)	16.0 (11.7–17.2)	–	0.25 (0.25–2.55)	0.25 (0.25–0.25)*	0.021	n.t.	0.002	<0.001

Concentrations are reported as medians (Q1–Q3). P, Mann–Whitney U-test.

*All measurements below the LLOQ.

LLOQ, lower limit of quantification; n.t., not tested.

The median testosterone, DHT and oestradiol concentrations were 116, 21 and 64 times higher in donor plasma compared with female infant plasma, respectively (figure 1A–C). There were no significant concentration differences between donor plasma and male infant plasma for testosterone and DHT, while the median concentration of oestradiol was 64 times higher in donor plasma than in the infant males.

Among the sex steroids without a gender difference before transfusion start, the concentrations of progesterone (figure 1D), DHEA (figure 1E) and androstenedione (figure 1F) were significantly higher in infant plasma compared with donor plasma, while oestrone (figure 1G) was lower in infant plasma (table 1).

### Sex steroid levels in infant plasma after transfusion

To investigate the impact of the plasma transfusion on infant sex steroids, we compared steroid levels in infant plasma before and after completed transfusion. No significant changes in any of the analysed sex steroids were observed in relation to the transfusion (table 2). Furthermore, there was no apparent influence of the transfusion on infant plasma sex steroid levels over the 72 hours following the transfusion (figure 1). No further changes in plasma steroids were observed in infants who received a second (n=8) or third (n=1) plasma transfusion during the study period (data not shown).

**Table 2:** Concentrations of sex steroids in infant plasma directly after completed plasma transfusion

Sex steroid	All infants (n=18)	Boys (n=7)	Girls (n=11)	P, all, pre vs post	P, boys, pre vs post	P, girls, pre vs post
Progesterone (pg/mL)	876 (700–1703)	990 (724–1728)	799 (642–1695)	0.349	n.t.	n.t.
Dehydroepiandrosterone (pg/mL)	6474 (2127–12 313)	6015 (1746–19 698)	6934 (2198–11 499)	0.943	n.t.	n.t.
Androstenedione (pg/mL)	1049 (740–1950)	1270 (916–1952)	1033 (688–1509)	0.215	n.t.	n.t.
Testosterone (pg/mL)	–	3471 (1742–11 456)	52 (4.0–70)	n.t.	0.866	0.484
Dihydrotestosterone (pg/mL)	–	266 (157–741)	16.0 (12.8–35.6)	n.t.	1.000	0.213
Oestrone (pg/mL)	4.6 (0.25–9.5)	8.2 (4.3–12.2)	2.2 (0.25–5.6)	0.650	n.t.	n.t.
Oestradiol (pg/mL)	–	0.25 (0.25–3.2)	0.25 (0.25–0.25)	n.t.	0.465	0.317

Concentrations are reported as medians (Q1–Q3). P, Wilcoxon signed-rank test.

n.t., not tested.

**Figure 2:**
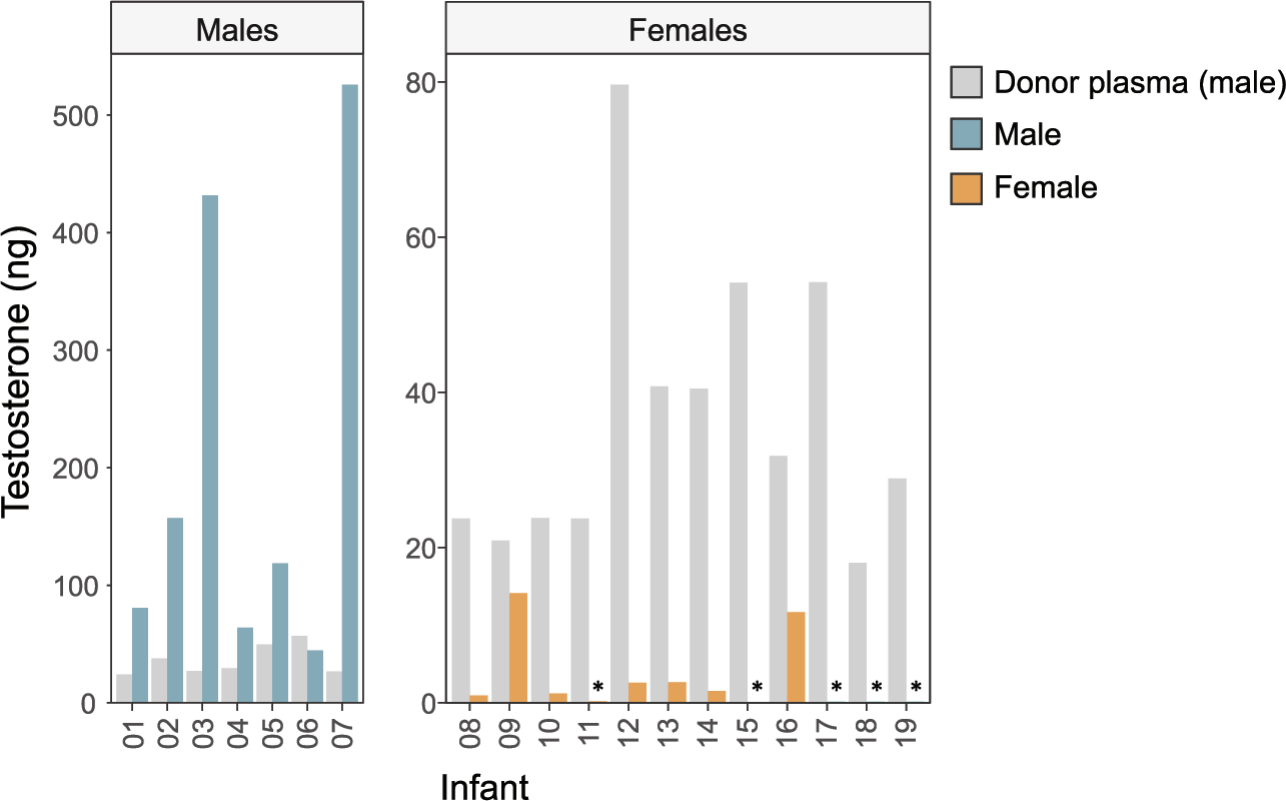
Total testosterone (ng) provided via the plasma transfusion (grey bars) in relation to circulatory amounts in male (blue bars) and female (yellow bars) infants. The total amount of testosterone provided via the plasma transfusion was calculated for each infant and compared with the total amount present in infant plasma before the start of the transfusion (assuming a volume of 50 mL/kg plasma in infants). *Plasma concentration below the LLOQ, therefore set to LLOQ/2=4 pg/mL. Note the different y-axis scales in males and females. LLOQ, lower limit of quantification.

As testosterone was the steroid showing the largest concentration difference between donor plasma and infant female plasma before transfusion, we further studied this hormone. Figure 2 shows the total amount of testosterone given via the transfusion compared with the total approximated amount present in infant plasma before the transfusion. In male infants, the amount of plasma testosterone was up to 20 times higher than that given via the transfusion. In female infants, testosterone provided via the transfusion was up to 600 times higher than that present in endogenous plasma.

### Influence of adult plasma on infant SHBG levels

**Figure 3:**
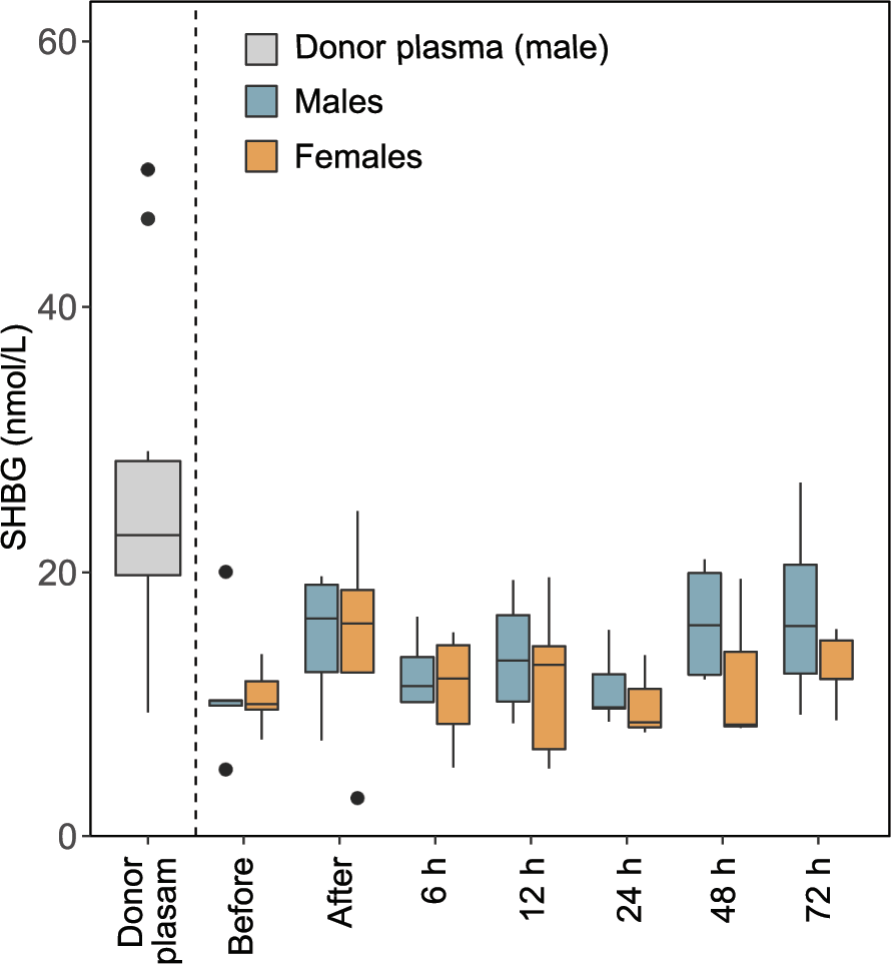
Effect of plasma transfusion on infant plasma levels of sex hormone-binding globulin (SHBG). SHBG was analysed in donor and infant plasma. Infant plasma samples were collected just before and after the completed plasma transfusion, and then followed 72 hours after the end of the transfusion.

Levels of SHBG were determined in a subpopulation of the cohort (five males and five females). The plasma SHBG concentration did not differ between genders before transfusion start (p=0.92, figure 3A). The SHBG concentration was significantly higher in donor plasma (median (Q1–Q3) 22.8 (17.1–33.5) nmol/L) compared with infant plasma (10.2 (9.1–12.3) nmol/L) before transfusion start (p=0.01). Among the few infants who had both a before and an after transfusion sample (n=8), there was a non-significant increase in plasma SHBG following the transfusion (10.2 (8.0–13.3) to 16.2 (9.1–19.5) nmol/L, p=0.21).

## Discussion

The results show that male adult donor plasma and very preterm infant plasma differ considerably in their sex steroid composition and concentrations. The largest differences were observed between donor and female recipients, particularly among the androgens. Nevertheless, our results indicate that a single plasma transfusion does not impact circulating levels of sex steroids in the recipient preterm infant.

The mean pre-transfusion level of progesterone was within the reference range at postnatal day 1 for infants born at 23–27 weeks’ gestation (2.9 nmol/L compared with 95% reference interval 1.9–11.5 nmol/L).^18^ Also the pre-transfusion levels of androstenedione and testosterone were within the reference intervals for very preterm infants.^19^ The oestradiol concentration in infant plasma was lower than in donor plasma and in most infant samples also below the LLOQ at 0.5 pg/mL, which translates to 1.8 pmol/L. However, Greaves *et al* 18 reported oestradiol 95% reference interval at day 1 to be 132–523 pmol/L for infants born at 23–27 weeks’ gestation. Also Trotter *et al* 20 reported substantially higher plasma oestradiol in extremely preterm female infants in the first week of life than measured in the current study. While we used GC-MS to analyse steroids, Greaves *et al* and Trotter *et al* used immunoassays, which likely contributes to the discrepancy in oestradiol concentrations between studies.

The concentrations of testosterone and DHT were unchanged in females after the transfusion although the male donor plasma provided up to 1000 times more of the steroids than present in the infant plasma. Similarly, oestradiol levels were unaffected by the transfusion, although concentrations were on average at least 30-fold lower in both preterm males and females compared with donor plasma. The lack of an effect of the transfusion on infant plasma steroids has several (mutually non-exclusive) possible explanations: that the provided steroids are rapidly cleared from circulation, metabolised to other products in the infant and/or instantly accumulated to peripheral tissues, for example, in the adipose tissue, and therefore not detectable in the plasma. Measuring infant urinary steroids and their metabolites may shed further light on the fate of the steroids delivered via plasma transfusions.

The transfusion did, on average, deliver 52 ng/kg testosterone to the infant. When postmenopausal women were treated with a single dose of 100 µg inhaled testosterone (corresponding to approximately 1.5 µg/kg), peak serum levels were observed minutes after the administration and then fell to pre-dose levels over the following 300 min.^21^ In that study, the initial half-life of total testosterone in serum was 10.1 min. Similar results in testosterone pharmacokinetics have been reported after sublingual administration and intravenous infusion of testosterone to women.^22 23^ The lack of an increase in plasma testosterone in female infants after plasma transfusion, with a relatively low dose from the donor plasma provided over 1–3 hours, indicates a blood clearance in accordance to that observed in adults. Furthermore, the relatively slow decline of androgens among males suggests that endogenous production remains significant in the first week of life.

Unconjugated steroids, as measured in this study, are hydrophobic molecules that readily diffuse across cellular membranes.^24^ Animal data support tissue uptake of intravenously supplied free steroids and a longer half-life in the tissues compared with blood.^25–29^ For example, Bonsall and Michael^29^ administered [^3^H]-testosterone to gonadectomised macaques 1 week after birth and examined sex steroid radioactivity in brain regions and peripheral tissues after 60 min. Both [^3^H]-labelled oestradiol and testosterone were detected in different brain regions. In contrast to steroids found in central and peripheral organs, low sex steroid radioactivity was detected in blood 60 min after administering [^3^H]-testosterone. Similar experiments in fetal macaques also support brain uptake of exogenously administered [^3^H]-testosterone.^28 29^ In neonatal rats, both non-selective and selective uptake of oestradiol into specific brain regions after intravenous administration of [^3^H]-oestradiol have been described.^30^ The [^3^H]-oestradiol concentration was 3–8 times higher in most brain regions and 80 times higher in the pituitary gland relative to blood after administration. Similarly, plasma infusion with oestrone to fetal sheep significantly enriches the steroid in several fetal brain regions.^31^ Based on these animal studies, it is plausible that excess sex steroids provided via adult plasma transfusions are redistributed and possibly also concentrated into other organs including the brain of the neonate.

Transient virilisation has been reported among girls born very preterm. In some cases, the virilisation has been associated with high androgen levels but with unclear aetiology.^32 33^ Repeated male blood transfusions have previously been suggested to be a cause of clitoromegaly.^16^ Our data show that male sex steroids are provided in high quantities with plasma transfusion, but do not provide evidence for a transient increase in serum androgens in association with the transfusion. A possible role for plasma transfusion in female virilisation needs further studies.

Only 1%–2% of the unconjugated steroids in circulation exist in the free active form. The large fraction of plasma steroids is instead bound to SHBG and albumin, and the bioavailability of sex steroids is thought to depend on the concentration of SHBG (the so-called *free hormone hypothesis*).^34 35^ Expression of human SHBG in castrated mice prolongs the half-life of circulatory DHT and testosterone but restricts steroid entry into target tissues.^36^ Thus, a change in plasma SHBG may affect the fraction of bioavailable steroids. SHBG levels in the donor plasma were about two-fold higher than in infants before the transfusion. Although infant plasma SHBG increased by 36% in response to the transfusion, the change was not statistically significant. Due to the low number of infants included in this analysis, these results must be interpreted with caution and should be investigated in a larger material.

This study has limitations. First, our data indicate that a higher temporal sampling resolution is required to follow the pharmacokinetics of transfusion-supplied steroids. However, additional blood sampling in this fragile patient group may not be ethically justifiable. Second, we only measured circulatory steroid levels and could not follow their distributions to tissues and possible endocrine effects. Third, we determined total plasma steroids, that is, the sum of the free bioavailable and the SHBG-bound fractions. There is evidence that the free fraction of testosterone (and other steroids) is most clinically relevant.^37^ However, determining free steroid levels is difficult, particularly in small volumes such as those available in this study, and most assays suffer from imprecision and inaccuracy. Among the strengths of this study is the simultaneous determination of a panel of sex steroids in donor and infant plasma using a sensitive GC-MS method.

## Conclusions

We conclude that sex hormones delivered via plasma transfusions from adult male donors do not significantly impact the circulating plasma concentrations in the preterm infant, irrespective of gender. The metabolic fate of the donor plasma steroids in the preterm infant and their possible effect on peripheral tissues and organ systems need further investigation. A first step in elucidating this will be to determine whether sex steroids provided via transfusion are subjected to urinary excretion in the infants.

## Data Availability

Data are available upon request.
