# Supplementary material for: Preterm infant circulating sex steroid levels are not altered by transfusion with adult male plasma: a retrospective multicentre cohort study
Source: Arch Dis Child Fetal Neonatal Ed. 2022 Mar 1;107(6):577–82. doi: 10.1136/archdischild-2021-323433 (PMC9606499; doi:10.1136/archdischild-2021-323433)
Supplement: Supplementary data [file fetalneonatal-2021-323433supp001.pdf]

Supplementary Table 1. Number of plasma samples analyzed for sex steroids according to time point and gender.

	**Before transfusion**	**After transfusion**	**6 h post transfusion**	**12 h post transfusion**	**24 h post transfusion**	**48 h post transfusion**	**72h post transfusion**
Males	7	7	7	6	7	7	7
Females	12	11	12	10	11	12	11
Donor plasma units	19						

Nilsson AK, *et al. Arch Dis Child Fetal Neonatal Ed* 2022; 107:577–582. doi: 10.1136/archdischild-2021-323433

Supplementary Table 2. Clinical characteristics of infants (n=19) included in the study.

**Variable**	**Value**
Male	7 (37%)
Gestational age (weeks)	25.3 (1.9)
	24.9 (22.9, 28.9)
Males	25.1 (1.1)
	24.9 (23.9, 26.9)
Females	25.5 (2.2)
	25.1 (22.9, 28.9)
Birth weight (g)	752 (208)
	680 (450, 1205)
Males	764 (136)
	775 (550, 915)
Females	744 (245)
	633 (450, 1205)
Time to first plasma transfusion (h)	34 (28)
	28 (6, 112)
Duration of first plasma transfusion (min)	132 (47)
	120 (60, 240)
Plasma transfusion volume (ml/kg)	9.9 (2.8)
	9.0 (6.0-15.0)
Infants receiving a second transfusion	8 (42%)
Infants receiving a third transfusion	1 (5%)

For categorical variables n (%) is presented.

For continuous variables Mean (SD) and Median (Min; Max) are presented.
